# Friction, Abrasion and Crack Growth Behavior of In-Situ and Ex-Situ Silica Filled Rubber Composites

**DOI:** 10.3390/ma13020270

**Published:** 2020-01-07

**Authors:** Sankar Raman Vaikuntam, Eshwaran Subramani Bhagavatheswaran, Fei Xiang, Sven Wießner, Gert Heinrich, Amit Das, Klaus Werner Stöckelhuber

**Affiliations:** 1 Leibniz-Institut für Polymerforschung Dresden e. V., Hohe Straße 6, 01069 Dresden, GermanyXiang@ipfdd.de (F.X.); wiessner@ipfdd.de (S.W.); gheinrich@ipfdd.de (G.H.); das@ipfdd.de (A.D.); 2 Fakultät Maschinenwesen, Technische Universität Dresden, 01062 Dresden, Germany; 3 Department of Materials ScienceTampere University, Korkeakoulunkatu 16, 33101 Tampere, Finland

**Keywords:** elastomers, in-situ silica, friction, abrasion, tear fatigue test

## Abstract

The article focuses on comparing the friction, abrasion, and crack growth behavior of two different kinds of silica-filled tire tread compounds loaded with (a) in-situ generated alkoxide silica and (b) commercial precipitated silica-filled compounds. The rubber matrix consists of solution styrene butadiene rubber polymers (SSBR). The in-situ generated particles are entirely different in filler morphology, i.e., in terms of size and physical structure, when compared to the precipitated silica. However, both types of the silicas were identified as amorphous in nature. Influence of filler morphology and surface modification of silica on the end performances of the rubbers like dynamic friction, abrasion index, and fatigue crack propagation were investigated. Compared to precipitated silica composites, in-situ derived silica composites offer better abrasion behavior and improved crack propagation with and without admixture of silane coupling agents. Silane modification, particle morphology, and crosslink density were identified as further vital parameters influencing the investigated rubber properties.

## 1. Introduction

Rubber is one of the most important polymer materials playing an essential role in dynamic applications like tires, belts, diaphragms, and seals. Rubber products are continuously subjected to repeated cyclic deformation, according to the service conditions. In most areas, due to the working conditions like rubbing, abrading, chunking, and tearing the product undergoes mechanical failure very easily. Such failure mainly occurs due to continuous friction, wear, abrasion, and crack formation. Friction and wear often occurs when two surfaces contact each other [1]. Movements between the two contacting surfaces cause cutting or damage of the rubber surface, usually known as abrasion [2]. The continuous cutting of the soft rubber material generates small cracks, which propagates faster when the rubber product is subjected repeatedly to dynamic working conditions. The mechanical performance of rubber composites depends on several material parameters like type of elastomers, type of filler, filler content, filler size and structure, state of filler dispersion, interaction between polymer and filler, type of plasticizer, and the nature of crosslinks and its density [3]. Natural rubber is one of the main rubbers used in truck tire applications due to its excellent self-mechanical reinforcement and crack growth resistance, which are attributed to its strain induced crystallization behavior [4,5]. Incorporation of fillers like carbon black and precipitated silica into the natural rubber further improves the reinforcement characteristics. In recent years, novel nanofiller systems such as layered silicates, carbon nanotubes, or graphene nanoplatelets have been emerging, which, due to their nanoscale and their special aspect ratios, offer reinforcements beyond carbon black or silica [6,7,8,9,10,11,12]. Non-crystallizable rubbers like styrene butadiene rubber and acrylonitrile butadiene rubber have no self-reinforcement behavior. Therefore, the rubbers display poorer tear strength and crack propagation characteristics. These synthetic rubbers do not have the ability to crystallize during mechanical deformation [13]. Technically, for such non-crystallizable rubbers, the reinforcement is mainly enhanced only via a proper selection of fillers [14,15,16]. 

Carbon blacks are the major class of reinforcing materials for rubbers fulfilling the requisite properties for more than a century due to its ability to offer outstanding mechanical performance [17,18]. However, in the last few decades, precipitated silica is emerging as a potential candidate and has partially or fully replaced carbon black, mainly due to lower rolling resistance and improved wet grip properties in tire applications despite sensitively varying tear strength values and crack resistance properties [16,19]. However, poor nano/microscale dispersion and interaction with rubber is one of the critical issues encountered with silica and is strongly influencing the failure properties of rubber products. However, these issues have been overcome through multi-stage mixing and by incorporating suitable silane coupling agents. Scientifically, several assumptions are believed about the rubber product failure when rubbers are subjected to the mentioned severe working conditions. Some of them are: (1) molecular chains attempt to move in the stressed direction, causing chains slippage and reorientation, (2) crosslinks, entanglements, and filler particles hinder chain motion, which leads to a state of tension and local chain scission occurs, (3) scission of single polymer chain transfers the load to the neighboring chains, which propagates and the cumulative effect produces microvoids, and, lastly, (4) the microvoids grow to from microcracks, which is an irreversible process and these microcracks propagate and, ultimately, cause product failure [19].

For the current study, silica filled rubber composites are prepared by two different methods. First, commercial precipitated silica is directly incorporated into the rubber and next silica particles are generated inside the rubber by the sol-gel method [20]. More details concerning the preparation of sol-gel silica inside the rubber could be found in our earlier work [21]. The volume fraction of silica in both nanocomposites is the same and is confirmed through thermogravimetric analysis [21]. The particle morphology (size and structure), crosslink density, and state of silica dispersion of the prepared SSBR composites are entirely different. The synthesis of in-situ silica in the polymer matrix leads to trapping of polymer chains inside the silica aggregates as well as strong linkage of the polymer molecules at the silica surface. This can be considered as additional network nodes and is responsible for enhanced reinforcement of the rubber without a silane coupling agent. Usage of silane results in a further increase of the physical properties of the composites with in-situ silica due to improved silica-rubber interaction. Incorporation of a masterbatch containing in-situ silica particles leads to commercial precipitated silica to (a) enhanced silica dispersion, (b) possible higher silica loading in the elastomer matrix without impairing its processability, (c) the possibility to control and customize the particle size, and (d) energy-effective processing due to easy incorporation of the filler particles. 

The main interest is to study how the filler size, structure, and crosslink density of the two different silica filled vulcanizates contribute to friction, abrasion, and crack propagation properties.

## 2. Materials and Methods

### 2.1. Materials

Solution styrene butadiene rubber (SSBR) BUNA 2525-2 VSL HM containing 25% vinyl content and 25% styrene content was supplied by Lanxess, Cologne, Germany. Tetrahydrofuran (THF), N-butyl amine, sulfur, zinc oxide, and stearic acid were acquired from Acros Organics, Germany. The vulcanizing accelerators *N*-cyclohexyl 2-benzothioazolesulfonamide (CBS-Vulkacit CZ) and diphenyl guanidine (DPG) were delivered by Lanxess, Germany. The precursor for the in-situ silica generation tetraethoxyorthosilicate (TEOS) was obtained from Sigma Aldrich (Munich, Germany) with a purity of 90%. The precipitated silica (Ultrasil VN-3) exhibiting a BET surface of 175 m^2^/g and a purity of 99% and the silane coupling agent bis[3-(triethoxysilyl)propyl]tetrasulfide (TESPT) were kindly provided by Evonik Industries, Essen, Germany. 

### 2.2. Preparation of Silica Rubber Composites

**Scheme 1:**
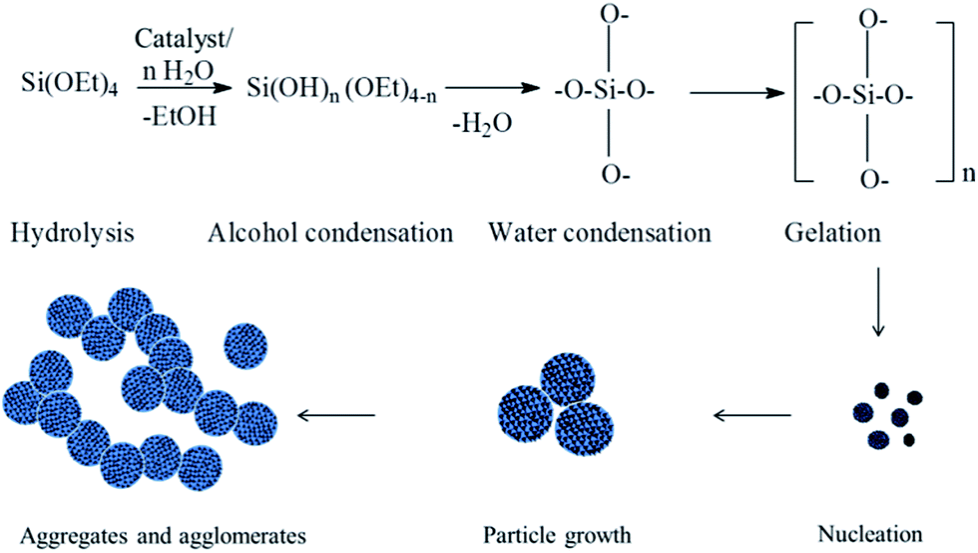
Silica particle formation through sol–gel reaction reprinted from Reference [20] with permission from the Royal Society of Chemistry.

The preparation of the in-situ SSBR silica composites was performed in two separate steps. In the first step, in-situ silica-rubber masterbatches were produced and, in the second one, this polymer-silica masterbatch was mixed with the other rubber chemicals. For preparing masterbatches, 30 g of SSBR was dissolved in 300 mL of THF and, then, 0.1 mole of TEOS and 0.2 moles of water were added into a round flask. After forming a standard mixture, 0.025 moles of n-butylamine catalyst were added and the homogeneous solution was stirred at 60 °C for 4 h. The viscous liquid obtained was treated in an ultrasonic bath for 10 min to prevent any pre-agglomeration of silica particles inside the rubber. This solution was then carefully given into 900 mL of ethanol. This process immediately solidifies and precipitates the rubber mass. The precipitate was filtered and dried for two days at 40 °C in a circulating air oven. Using different amounts of TEOS and water (always at the molar ratio 1:2) leads to a controlled variability of volume fractions of silica in the rubber. Scheme 1 shows the chemical reaction for the formation of the silica particles and the particle growth [20]. 

The second stage of preparation of in-situ silica composites consists of two sub-steps. In the first sub-step, in an internal mixer (HAAKE Rheomix 600P internal mixer, Karlsruhe, Germany), few grams of raw SSBR are added to the dried in-situ silica SSBR masterbatch. This is done to fine-tune the silica concentration in rubber and also to achieve the desired fill factor of 0.7 (70% filling). After adequate thorough blending, other rubber components as zinc oxide (2 phr), stearic acid (3 phr), and the TESPT coupling agent were added sequentially. This compounding process was carried out at 110 °C and at 80 rpm for 6 min. The mixture was dumped at a temperature of ~140 °C. In the second sub-step, the vulcanizing chemicals CBS (1.4 phr), DPG (1.7 phr), and sulfur (1.4 phr) were mixed into the compound in a two roll mill (Polymix-110L, Servitec, Wustermark, Germany) at 50 °C for 10 min with a constant friction of a 1:1.2 ratio.

Likewise, commercial precipitated silica composites were prepared by the incremental addition of zinc oxide, stearic acid, silica (Ultrasil-VN3), and silane TESPT into the SSBR rubber in the internal mixer. Mixing was carried out at 110 °C and 80 rpm for 10 min. For the addition of vulcanization chemicals in the two-roll mill, a process similar to that used for in-situ silica was applied. The finished compounds of in-situ and precipitated silica were then allowed to age for 24 h at room temperature. The stored rubber compounds were rheometrically analyzed in a rubber process analyzer (Scarabaeus SIS-V50, Scarabaeus GmbH, Wetzlar, Germany) to determine their optimal vulcanization time TC_90_. Vulcanization of these rubber samples was conducted at 160 °C into sheets of 2-mm thickness in a heated compression molding press (Fontijne TP1000, Delft, The Netherlands) with a force of 150 kN for optimum vulcanization time TC_90_ plus 1 min per mm thickness of the sample. The prepared silica-rubber samples are abbreviated as i-30, i-30s, x-30, x-30s. ‘i’ and ‘x’ represents in-situ and commercial precipitated silica filled composites, respectively. The number refers to the amount of silica in rubber (mentioned in ‘phr’—parts per hundred rubber) and the suffix ‘s’ after the number denotes rubber composites containing a TESPT silane coupling agent.

### 2.3. Characterizations of Silica-SSBR Nanocomposites

#### 2.3.1. X-ray Diffraction (XRD)

X-ray diffraction analysis was carried out by means of a 2-circle diffractometer XRD 3003 T/T (Seifert-FPM, Freiberg, Germany) with Cu-Kα radiation at 30 mA and 40 kV from 2θ = 1° to 16° using 0.05° as the step length.

#### 2.3.2. Dynamic Light Scattering Analysis (DLS)

The particle size and particle distribution of the silica powders was determined with a dynamic light scattering analyzer (Dyna Pro-nanostar, Wyatt instruments, Santa Barbara, CA, USA) from 0.2 to 2500 nm, using a laser wave length of 785 nm. Measurements are performed at 25 °C with dispersions of silica particles in ethanol. The DLS experiments were carried out for three samples. At least 25 scans per sample were performed.

#### 2.3.3. Scanning Electron Microscopy (SEM)

Using an Ultra plus electron microscope from Carl Zeiss NTS GmbH, Oberkochen, Germany (3 kV, 30 µm aperture size, and SE2 detector), scanning electron microscope images of the silica-elastomer composites were recorded. Strip samples of these elastomer composites were broken after being exposed to liquid nitrogen. The respective fracture surface was then sputtered with 3-nm platinum with the BAL-TEC SCD 500 Sputter Coater and investigated under SEM at a zero angle.

#### 2.3.4. Linear Friction Tester

**Figure 1:**
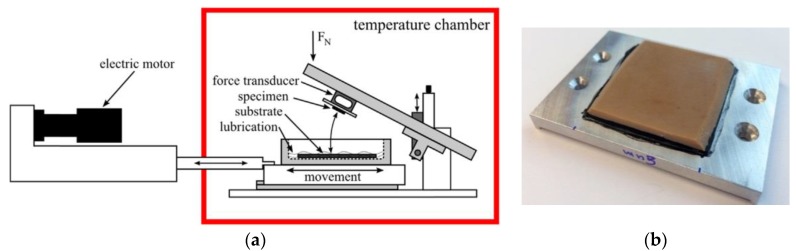
(**a**) Setup of the friction tester (schematically) reprinted from Reference [22] with permission from Elsevier and (**b**) an example test specimen.

Experiments on the friction properties of the SSBR-silica composites were carried out, using a tribometer-linear friction tester, located at the Deutsches Institut für Kautschuktechnologie (DIK), Hannover, Germany [22]. The instrument is shown schematically in Figure 1a. The test samples 20 mm × 20 mm × 10 mm by size were prepared in the compression molding machine (Fontijne TP1000, Delft, The Netherlands) at 160 °C at a force of 150 kN. The edges of the specimen are chamfered to avoid kinking of the elastomer specimen on contact with the asphalt surface. An example of the test specimen is shown in Figure 1b. Measuring temperatures from 2 °C to 100 °C, sliding velocities from 0.1 mm/s to 300 mm/s, and a variation of the load from 1 bar to 7 bar are possible at this experimental setup. Current experiments were performed at 15 °C with a load of 2 bars at velocities 0.1 mm/s to 300 mm/s. Measurements were performed on fine asphalt surface wetted with water to mimic wet friction properties of rubber composites similar like wet skid behavior of tires under ABS-braking conditions. It is commonly assumed that the ABS wet braking temperature for tire tread compound is ca. 40 °C (i.e., usually somewhat larger as the ambient temperature in mid-Europe) and the slipping velocity is around 1 m/s. It should be noted that, for the vehicle friction, the tread block slip velocity depends on time. In other papers, an effective (say time-averaged) slip velocity *v* = 0.3 m/s in water and *v* = 0.1 m/s in the dry state [23] was presumed. In reality, however, the tread blocks experience an inhomogeneous slip dynamic, where the slip velocity is near zero when a tread block enters the tire road footprint, while the slip velocity is typically in the order of a few m/s when the tread block is retracted from the footprint. The friction tester in our experiments is limited to velocities lower than 1 m/s. For this, we used the time-temperature superposition principle (Williams–Landel–Ferry (WLF) equation) to calculate the correct temperature for 10 mm/s (the measurable velocity of the machine). Therefore, the test temperature was determined using the universal WLF constants (*C*_1_ = 17.44 and *C*_2_ = 51.6 K) with help of the glass transition temperature of composites (*T_g_*). The *T_g_* values of the unfilled rubber as well as the silica-filled composites were determined by dynamic mechanical measurements (temperature sweep mode at a frequency of 10 Hz) [20].

Derived from the *T_g_* of the elastomer compounds and the WLF principle, the temperature *T_calc_* was calculated to be ca. 15 °C for a velocity of 10 mm/s (see Table 1). This temperature *T_calc_* was further used in our experimental measurements.

#### 2.3.5. Abrasion Test

The abrasion experiments were conducted in a rotary drum abrasion tester (Bareiss Prüfgerätebau GmbH, Oberdischingen, Germany, DIN ISO 4649 [24]) using 10 N static load. Circular samples with dimensions ∅16.1 mm × 4.2 mm were tested. The abrasion resistance index (*ARI*) was calculated by the standard given formula in Equation (1).

(1) $$ARI=\frac{\Delta m_{r}\times \rho_{t}}{\Delta m_{t}\times \rho_{r}}\times 100,$$

where $\Delta m_{r}$ is the loss of mass from the standard rubber test specimen #1 in mg, $\rho_{r}$ is the density of the standard elastomer sample #1 in g/cm^3^, $\Delta m_{t}$ is the mass loss of the test rubber piece in mg, and $\rho_{t}$ is the density of the elastomer test sample in g/cm^3^.

#### 2.3.6. Tear Fatigue Analyzer

**Figure 2:**
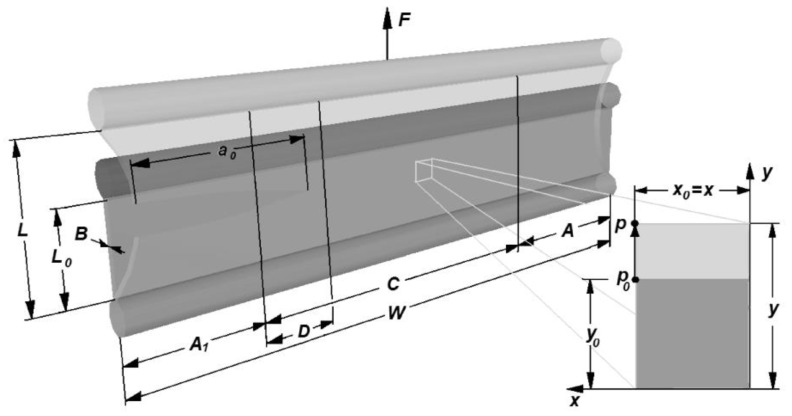
Schematic representation of a pure shear sample with depiction of the regions having different stress conditions (according to Reference [25]).

The fracture mechanics behavior of the rubber-silica composites was investigated using an Instron Electroplus E1000 (Darmstadt, Germany). Dumbbell-shaped pure shear specimens with length *L*_0_ = 10 mm, thickness *B* = 1 mm, and width *W* = 80 mm (see Figure 2) were used to measure the crack propagation in these rubbers. The notch for crack initialization was made by hand for a length *a*_0_ of 25 mm on one side of the pure shear sample, which generates a crack of the length *a*, and is expected to be adequately long related to the length (*L*_0_) of the specimen. The crack propagation measurements were performed at room temperature (23 °C) using 1 N pre-force. Details of the experiments can be found in literature [25,26,27,28]. Rivlin and Thomas [29] stated that the tearing energy is the specific parameter of fatigue crack propagation in elastomers, which is defined as $T=-{\left(\delta W/\delta A\right)}_{L}$. Hereby, *W* is the elastic strain energy and *A* is the interfacial area of the propagated crack. Based on the assumption of Rivlin and Thomas [29], the pure shear specimen can be divided into four regions (see Figure 2). The elastomer in area ***A***_1_ is not strained. In region *D*, complicated deformation stress and strain fields occur due to the tip of the crack. In the *C*-region, pure shear deformation can be found and there is a region designated as *A*, where the edge effects occur. The deformation state in region *D* does not change but shift parallel in the crack growth direction, when the crack length increases by *da*. Then, the volume of the *C*-region is equal *L*_0_ × *B* × *da* and is, therefore, smaller and the stored elastic energy in this volume is released. Samples with pure shear geometry are used because, hereby, the tearing energy *T_p_* does not depend on the crack length.

(2) $$T_{P}=w\cdot L_{0}$$

Hereby, *w* is the elastic pure shear strain-energy density, measured from the unloading cycle of the stress-strain curve of an un-notched sample. Two un-notched pure shear specimens with diverse widths (*W*_1_ = 80 mm and *W*_2_ = 60 mm) were employed for evaluating the pure shear elastic strain energy density *w* to eliminate the energy contribution of the edge effect region.

(3) $$w=\frac{U_{1}-U_{2}}{\left(W_{1}-W_{2}\right)\cdot L_{0}\cdot B}$$

where *U*_1_ and *U*_2_ are the elastic strain energies for the two different sample widths *W*_1_ and *W*_2_, respectively.

Paris and Erdogan [30] describe the correlation between a stable fatigue crack propagation rate da/dn and the tearing energy by a power law equation.

(4) $$da/dn=\beta \cdot T^{m}\;\mathrm{for}\;T_{1}\leq T\leq T_{c},$$

β and *m* are, hereby, constants of the material. *T*_1_ represents the beginning of the stable crack growth regime and *T_c_* is the critical tearing energy, which characterizes the changeover to an unstable regime.

## 3. Results and Discussion

### 3.1. Morphology

**Figure 3:**
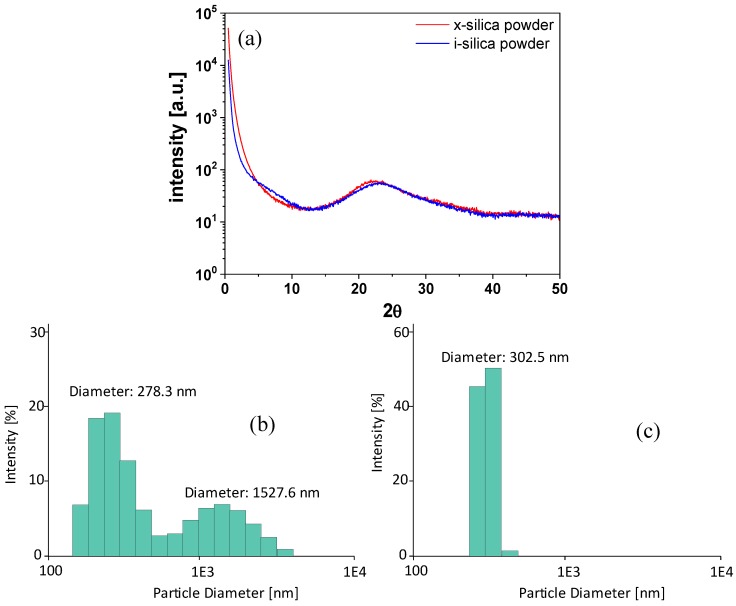
(**a**) X-ray diffraction patterns of in-situ silica and precipitated silica powders. Particle size histograms of (**b**) precipitated silica (Ultrasil-VN3) and (**c**) in-situ silica, from dynamic light scattering measurements.

Figure 3a displays the x-ray diffraction analysis (XRD) of in-situ and precipitated silica powder samples. The broad XRD band is located in the 2θ region of 15° to 35°. This confirms that the physical state of both silica types is entirely amorphous and there is no significant difference in the crystalline structure.

A dynamic light scattering measurement (DLS) was performed to characterize the two silica types in particle size (hydrodynamic diameter) and morphology. The precipitated silica powder particles (Ultrasil VN3) in Figure 3a display a bimodal distribution with a wide spectrum of different particle sizes. These silica particles are aggregated and agglomerated. The aggregate and agglomerate diameter sizes range from 150 to 1500 nm. The DLS results for the in-situ derived silica particles (see Figure 3b) give an average diameter of around 200 to 500 nm. The in-situ silica particles exhibit a mainly monodisperse or unimodal narrow particle distribution.

**Figure 4:**
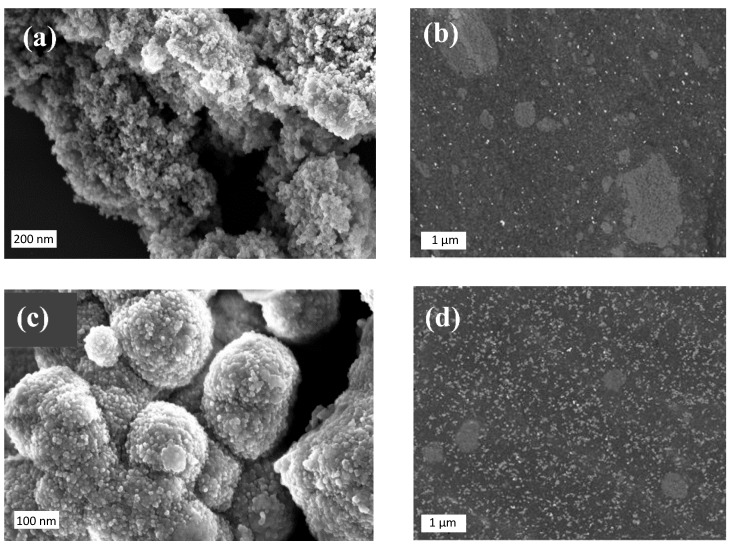
SEM images of (**a**) pristine commercial precipitated silica powder, and (**b**) its respective SSBR composite. (**c**) In-situ silica powder (extracted from un-crosslinked rubber) and (**d**) its respective SSBR composite.

SEM images of two silica powders and their respective SSBR rubber composites are given in Figure 4. Figure 4a shows how the precipitated silica particles look i.e., a network or chain-like structures with highly aggregated morphology. In Figure 4c, the in-situ silica particles display spherical aggregates with particle sizes from 200 to 400 nm. Nevertheless, the average primary particle sizes of both silica types appear to be in the order of ~10 to 15 nm. Figure 4b,d show the SEM pictograms of brittle fracture surfaces of 30 phr filled commercial precipitated silica and in-situ silica filled SSBR composites, respectively. The fractured images highlight the state of silica dispersion, which appears to be significantly different for either of the silica. An improved dispersion is found in the in-situ silica system.

### 3.2. Friction Coefficients μ and Friction Curves

**Figure 5:**
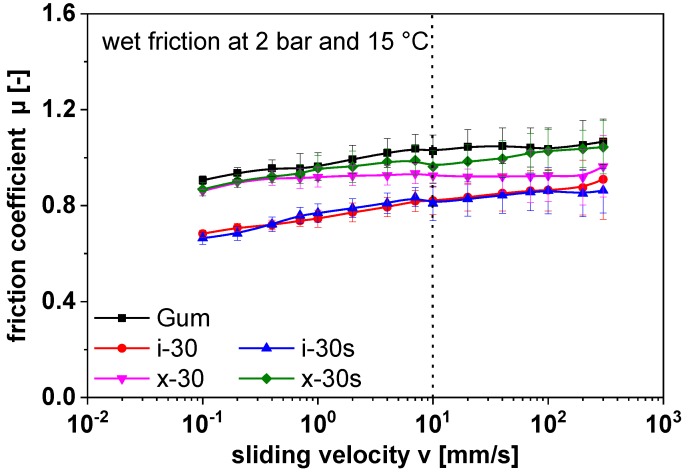
Friction behavior of unfilled rubber, in-situ silica, and precipitated silica with and without addition of a silane coupling agent. The dotted line corresponds to ABS wet braking conditions at 40 °C and a slipping velocity of 1 m/s.

Figure 5 shows the experimental friction results measured at different sliding velocities with a normal load of 2 bar. The experimental results show that the unfilled elastomer exhibits a higher friction coefficient. An incorporation of silica in SSBR reduces the friction coefficient slightly. Precipitated silica without any silane exhibits a lower coefficient of friction in comparison to their respective silane-modified systems. The application of silane coupling agents to the system filled with precipitated silica has significantly improved the friction behavior in the high velocity range. The in-situ silica-filled systems exhibited lower friction coefficient values when compared to the precipitated silica system. It should be noted that incorporation of the silane coupling agent into the in-situ silica system did not affect the friction behavior. This result supports our earlier findings that in-situ silica has the capability to reinforce the rubber matrix without the need of any silane [20,21,31]. For good ABS (anti-lock braking or anti-skid braking) wet braking, the friction coefficient of the rubber system must be higher (or nearly equal to 1). Therefore, from the analysis, samples are rated as per their wet traction performance: Gum > x-30s > x-30 > i-30s > i-30.

On the other hand, rubber composites must display a lower friction coefficient to achieve lower rolling resistance properties. Lower rolling resistance contributes to better fuel efficiency of the vehicle. Lower rolling resistance can be observed when the rubber compounds display lower hysteresis and smaller tan δ in the temperature range around ca 60 °C as well as with a reduced rolling friction. From this perspective, the in-situ silica system will offer better low rolling resistance [21]. The samples rated as per their low rolling resistance (larger the better): Gum < x-30s < x-30 < i-30s < i-30.

### 3.3. Abrasion Test

**Figure 6:**
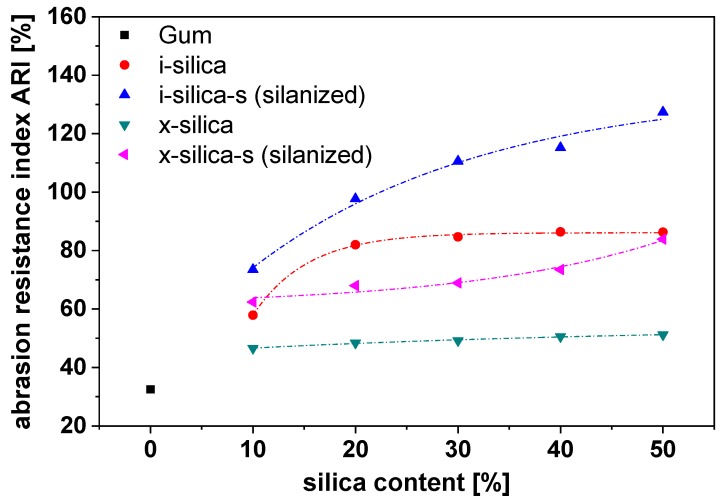
ARI of gum, in-situ, and precipitated silica compounds and its TESPT modified SSBR composites.

Figure 6 shows the abrasion resistance index (*ARI*) of the unfilled vulcanizate, in-situ silica and precipitated silica-filled and their respective silane modified composites. The unfilled gum vulcanizate exhibits least *ARI* and this is due to the poor physical properties of the gum rubber, where the rubber surface is easily cut-out by the abrasion forces. Addition of silica as reinforcing filler into the SSBR-rubber improves the *ARI* values. In addition, increasing the amount of silica in rubber gradually increases the *ARI* values. However, the improvements in *ARI* are not significant without the presence of silanes. In the case of precipitated silica without silane, no improvements could be seen in the ARI values beyond the addition of 30 phr of silica. The incorporation of TESPT as a coupling agent in the silica-rubber system improves the abrasion properties further in both in-situ and precipitated silica composites. Experimental observations indicate improved abrasion properties for the precipitated silica compounds in comparison to them with in-situ silica. The effective particle size and surface area of the commercial precipitated silica are higher than in-situ silica, which could be the reason for the improved abrasion characteristics.

**Figure 7:**
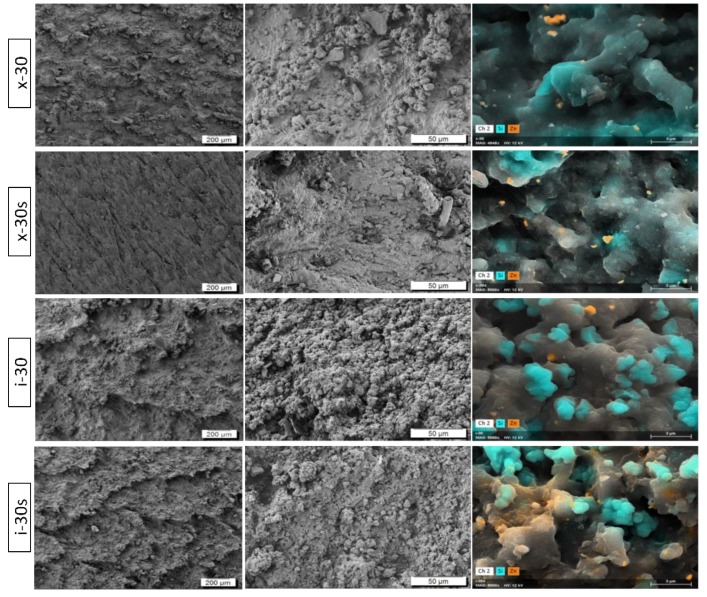
SEM and EDX images of abraded surfaces (after the DIN abrasion test) of 30 phr precipitated silica, in-situ silica, and their TESPT modified SSBR composites.

To understand the abrasion behavior in detail, the topology of abraded surfaces is investigated by SEM spectroscopy and their respective micrographs are given in Figure 7. The abraded surfaces of SSBR samples filled with precipitated silica and in-situ silica compounds without the coupling agent TESPT are found to be rough in comparison with the silane modified samples. Absence of silane as a coupling agent easily allows the rubber particles to cut-off due to the rubbing force. This is because of the high inhomogeneity in the elastomer material and the poor filler-rubber interaction. Silane modification of both silica composites greatly improves the surface wear mechanism. Formation of ridges or waviness protects the rubber surface from the further abrasion process by the gradual reduction of the surface contact area [32,33]. Such ridge topologies are formed on the surface of the samples when they are modified with a silane coupling agent. This also shows the improved interaction between silica and polymer assisted by the silane coupling. 

The lower ARI of the in-situ silica/SSBR composite could be attributed to the lower surface area and also due to the bigger particle size of the in-situ silica filler. In the EDX images in Figure 7, for samples without a silane coupling agent, it could be noticed that the silica filler particles are highly visible or exposed (noticeable as a blue color) after the abrasion experiment, since the silica particles are covered by a polymer layer. In in-situ silica-filled systems, the filler particles (noticeable as a blue color) are highly separated from the rubber matrix, when compared to the precipitated silica system. This is an outcome of the poor abrasion behavior of the in-situ silica. In our previous investigation, we observed that the specific surface area of in-situ silica is very low in comparison to a precipitated silica system [34].

### 3.4. Tear Fatigue Analyzer (TFA)—Crack Propagation Measurements

**Figure 8:**
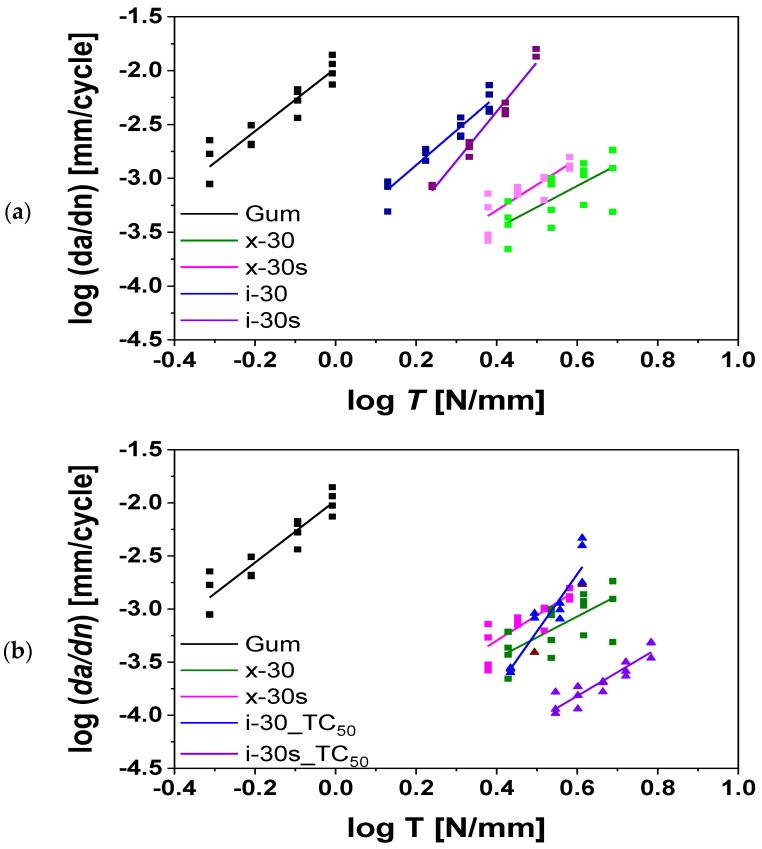
(**a**) Paris plots showing the crack propagation rate as a function of tearing energy for in-situ silica and precipitated silica-filled composites and (**b**) crack propagation of equally adjusted crosslink density of in-situ silica and precipitated silica compounds.

A tear fatigue analysis was performed to understand the crack propagation behavior of different silica-filled SSBR composites and the influence of silica surface modification by a silane coupling agent. A logarithmic plot of the crack propagation rate (*da*/*dn*) in relation to the tearing energy (*T*) is known as a Paris plot and is given for all the samples in Figure 8. The rate of crack growth (*da*/*dn*) is directly proportional to the tearing energy *T* of the material. Therefore, all the samples are tested inside the stable crack growth regime. In Figure 8a, as expected, the unfilled gum sample shows a smaller tearing energy and a quicker crack formation compared to filled samples. It means that there is no active material to prevent the crack propagation in the gum vulcanizate. Incorporation of both in-situ and precipitated silica significantly improves the tear fatigue behavior. The main mechanism is the hindrance of filler particles that resist the crack tip growth [3]. Interestingly, the crack propagation behavior is found to be significantly different for the in-situ and precipitated silica, possibly due to the differences in their morphologies. The silane modification improved the crack propagation behavior for both the silica fillers. It is plausible that the significantly better crack resistance in silane-containing systems is a result of the establishment covalent chemical bonds between the elastomer matrix and filler particles by means of the bi-podal sulfur containing silane molecules. However, the in-situ silica system shows a higher crack growth rate than the precipitated silica system. The results, therefore, show that the state of the silica dispersion is not only a critical parameter for crack propagation, but that particle size, particle morphology, cross-linking density, and filler-polymer interfaces also play an important role.

Our previous investigations of the SEM and TEM analysis distinctively states that particle size and morphology of precipitated and in-situ silica as well as the reinforcement characteristics are entirely different [20]. Moreover, the quantity of assessed cross-linking density is higher for the in-situ silica composites in comparison to precipitated silica composites. To eliminate the effect of different cross-linking density on the crack propagation behavior, the crosslink density of in-situ samples are reduced and matched with those of precipitated silica composites by modifying the state of cure from *TC*_90_ to *TC*_50_. The amount of cross-linking density was measured by means of the equilibrium swelling method and the Flory-Rehner equation, which is described in detail in our previous paper [20]. The values of the cross-linking densities from this measurement are given in Table 2. The crack propagation behavior is completely different after adjusting the crosslink density of the in-situ silica composites. The plots in Figure 8b clearly indicate that the crack propagation behavior of in-situ silica composites is significantly improved. The in-situ silica compounds without silane modification yield better properties than the precipitated silica samples. However, the incorporation of silane in both of the silica systems reduces the crack propagation properties by a certain amount.

## 4. Conclusions

The present work has discussed the influence of in-situ silica and precipitated silica on friction, abrasion, and crack growth properties of SSBR rubber. For better understanding of the significance of silane in the silica-filled elastomer systems, the TESPT silane coupling agent was incorporated. The silanization affected the friction characteristics (wet grip performance) of precipitated silica-filled rubber. However, no significant effect was observed in the in-situ silica systems. Lower friction coefficients were observed for the in-situ silica systems, which is very vital for low rolling resistance (better fuel efficiency) applications. The precipitated silica compounds displayed better abrasion characteristics and the incorporation of a silane coupling agent plays a significant role by improving the filler-rubber interaction. The crack propagation results initially indicated that the precipitated silica system has higher resistance to crack propagation. However, upon comparing samples with similar crosslink densities, much better resistance to crack growth was observed for the in-situ silica systems, which is further improved by an additional silane modification. As a consequence of the results described in this paper, it can be stated that rubber compounds using a masterbatch containing in-situ silica (instead of the classical silica/silane mixing process) may have the potential to be used in the future in the industrial production of technical rubber articles or car tires. Yet, further research efforts are required for this implementation, in particular to ensure the scale-up of the process to industrial dimensions.

## Figures and Tables

**Table 1:** Glass transition temperatures of elastomer compounds and the calculated temperatures for the friction measurement by using the WLF principle.

Sample	*T_g_*/°C	*T_calc_* Calculated Temperature/°C	Hardness Shore A	Crosslink Density/ 10^−4^ g/cm³
**Gum**	−27.8	14.96	42	1.463
**i-30**	−27.8	14.96	56	2.128
**i-30s**	−26.0	15.63	59	2.718
**x-30**	−28.2	14.81	56	1.571
**x-30s**	−26.8	15.33	57	2.202

**Table 2:** Tear fatigue behavior of SSBR compounds filled with different silica systems with a reference to crosslinking density.

Sample	Cross-Linking Density /10^−4^ mol/cm^3^	Slope
**SSBR-Gum**	1.463	2.9
**x-30**	1.571	3.2
**x-30s**	2.202	4.6
**i-30**	2.128	2.4
**i-30s**	2.718	1.9
**i-30_TC_50_**	1.677	2.2
**i-30s_TC_50_**	2.232	5.4

## References

[1] Persson B.N.J. (1998). On the theory of rubber friction. Surf. Sci..

[2] Schallamach A. (1958). Friction and Abrasion of Rubber. Rubber Chem. Technol..

[3] Persson B.N.J., Albohr O., Heinrich G., Ueba H. (2005). Crack propagation in rubber-like materials. J. Phys. Condens. Matter.

[4] Rooj S., Das A., Morozov I.A., Stöckelhuber K.W., Stoček R., Heinrich G. (2013). Influence of “expanded clay” on the microstructure and fatigue crack growth behavior of carbon black filled NR composites. Compos. Sci. Technol..

[5] Gent A.N., Lindley P.B., Thomas A.G. (1964). Cut growth and fatigue of rubbers. I. The relationship between cut growth and fatigue. J. Appl. Polym. Sci..

[6] Galimberti M. (2011). Rubber—Clay Nanocomposites.

[7] Bhowmick A.K., Bhattacharya M., Mitra S., Kumar K.D., Maji P.K., Choudhury A., George J.J., Basak G.C., Heinrich G. (2011). Morphology-property relationship in rubber-based nanocomposites—Some recent developments. Advanced Rubber Composites.

[8] Sadasivuni K.K., Ponnamma D., Thomas S., Grohens Y. (2014). Evolution from graphite to graphene elastomer composites. Prog. Polym. Sci..

[9] Bokobza L., Mittal V. (2014). Elastomers filled with carbon nanotubes. Polymer Nanotube Nanocomposites.

[10] Yaragalla S., Mishra R.K., Thomas S., Kalarikkal N., Maria H. (2019). Carbon-Based Nanofillers and Their Rubber Nanocomposites Fundamentals and Applications.

[11] Klüppel M., Möwes M.M., Lang A., Plagge J., Wunde M., Fleck F., Karl C.W., Stöckelhuber K., Das A., Klüppel M. (2016). Characterization and Application of Graphene Nanoplatelets in Elastomers. Designing of Elastomer Nanocomposites: From Theory to Applications.

[12] Najam M., Hussain M., Ali Z., Maafa I.M., Akhter P., Majeed K., Shehzad N. (2019). Influence of silica materials on synthesis of elastomer nanocomposites: A review. J. Elastom. Plast..

[13] Lake G.J., Lindley P.B. (1964). Cut growth and fatigue of rubbers. II. Experiments on a noncrystallizing rubber. J. Appl. Polym. Sci..

[14] Rattanasom N., Saowapark T., Deeprasertkul C. (2007). Reinforcement of natural rubber with silica/carbon black hybrid filler. Polym. Test..

[15] Dannenberg E.M. (1975). The Effects of Surface Chemical Interactions on the Properties of Filler-Reinforced Rubbers. Rubber Chem. Technol..

[16] Reincke K., Grellmann W., Heinrich G. (2004). Investigation of Mechanical and Fracture Mechanical Properties of Elastomers Filled with Precipitated Silica and Nanofillers Based upon Layered Silicates. Rubber Chem. Technol..

[17] Kraus G. (1978). Reinforcement of Elastomers by Carbon Black. Rubber Chem. Technol..

[18] Medalia A.I. (1978). Effect of Carbon Black on Dynamic Properties of Rubber Vulcanizates. Rubber Chem. Technol..

[19] Heinrich G., Horst T., Struve J., Gerber G. Crack propagation in rubber-like materials. Proceedings of the 11th International Conference on Fracture 2005 (ICF11).

[20] Raman V.S., Das A., Stöckelhuber K.W., Eshwaran S.B., Chanda J., Malanin M., Reuter U., Leuteritz A., Boldt R., Wießner S. (2016). Improvement of mechanical performance of solution styrene butadiene rubber by controlling the concentration and the size of in situ derived sol–gel silica particles. RSC Adv..

[21] Vaikuntam S.R., Bhagavatheswaran E.S., Stöckelhuber K.W., Wießner S., Heinrich G., Das A. (2017). Development of high performance rubber composites from alkoxide-based silica and solution styrene-butadiene rubber. Rubber Chem. Technol..

[22] Lang A., Klüppel M. (2017). Influences of temperature and load on the dry friction behaviour of tire tread compounds in contact with rough granite. Wear.

[23] Tolpekina T.V., Persson B.N.J. (2019). Adhesion and Friction for Three Tire Tread Compounds. Lubricants.

[24] DIN ISO 4649:2002 (2002). Rubber, Vulcanized or Thermoplastic—Determination of Abrasion Resistance Using a Rotating Cylindrical Drum Device.

[25] Stoček R., Heinrich G., Gehde M., Heinrich G., Kaliske M., Lion A., Reese S. (2010). The influence of the test properties on dynamic crack propagation in filled rubbers by simultaneous tensile and pure shear test mode testing. Const. Models Rubber VI.

[26] Stoček R., Heinrich G., Reincke K., Grellmann W., Gehde M. (2010). Einfluss der Kerbeinbringung auf die Rissausbreitung in elastomeren Werkstoffen. KGK Kautsch. Gummi Kunstst..

[27] Stoček R., Heinrich G., Reincke K., Grellmann W., Gehde M. (2011). Rissausbreitung in Elastomeren Werkstoffen unter dynamischer Beanspruchung: Einfluss der Kerbeinbringung. KGK Kautsch. Gummi Kunstst..

[28] Ghosh P., Stoček R., Gehde M., Mukhopadhyay R., Krishnakumar R. (2014). Investigation of fatigue crack growth characteristics of NR/BR blend based tyre tread compounds. Int. J. Fract..

[29] Rivlin R., Thomas A.G. (1953). Rupture of rubber. I. Characteristic energy for tearing. J. Polym. Sci..

[30] Paris P., Erdogan F. (1963). A critical analysis of crack propagation laws. J. Basic Eng..

[31] Raman V.S., Rooj S., Das A., Stöckelhuber K.W., Simon F., Nando G.B., Heinrich G. (2013). Reinforcement of Solution Styrene Butadiene Rubber by Silane Functionalized Halloysite Nanotubes. J. Macromol. Sci. Part A.

[32] Gent A.N., Pulford C.T.R. (1983). Mechanisms of rubber abrasion. J. Appl. Polym. Sci..

[33] Tabsan N., Wirasate S., Suchiva K. (2010). Abrasion behavior of layered silicate reinforced natural rubber. Wear.

[34] Vaikuntam S.R., Stöckelhuber K.W., Subramani Bhagavatheswaran E., Wießner S., Scheler U., Saalwächter K., Formanek P., Heinrich G., Das A. (2018). Entrapped Styrene Butadiene Polymer Chains by Sol-Gel-Derived Silica Nanoparticles with Hierarchical Raspberry Structures. J. Phys. Chem. B.

